# Association between internal migration experience and depressive symptoms: analysis of PSID data

**DOI:** 10.1186/s12889-023-16073-0

**Published:** 2023-06-14

**Authors:** Zi-Xuan Liao, Xiao-Min Tan, Ying-Ying Zhao, Xiao-Cui Sun, Fa-Ling Yi

**Affiliations:** 1 Guangdong Pharmaceutical University, No. 280 Waihuan East Road, University Town, Guangzhou, 510006 Guangdong Province China; 2 Engineering and Technology Research Center of Guangdong Universities—Real World Engineering and Technology Research Center of Medical Information, Guangzhou, 510006 China

**Keywords:** Internal migration experience, Depressive symptoms, Causes, Mental health

## Abstract

**Background:**

Depression is on the rise globally. Additionally, the United States has a high level of population mobility. The main aim of this study was to provide a reference for improving the mental health of internal migrants by investigating the relationship between internal migration experience and depressive symptoms.

**Methods:**

We analysed data from the Panel Study of Income Dynamics (PSID). We included PSID data from the 2005 to 2019 waves in which all respondents were asked about their internal migration experience and depressive symptoms. This study included 15,023 participants. T tests, chi-square tests, multiple logistic regression methods were performed and fixed effects model.

**Results:**

In the sample, the prevalence of depressive symptoms was 4.42%. The risk of depression in internal migrants was 1.259 times (OR = 1.259, 95% CI = (1.025–1.547, *p* < 0.05) that of nonmigrants. Internal migration experience was significantly positively associated with female depressive episodes (OR = 1.312, 95% CI = 1.010–1.704, *p* < 0.05) and increased risk of becoming depressed at a young age (OR = 1.304, 95% CI = 1.010–1.684, *p *< 0.05). The association between internal migration experience and depressive symptoms was more significant for participants who might move (OR = 1.459, 95% CI = 1.094–1.947, *p* < 0.05). In addition, different internal migratory causes are associated with depressive symptoms to varying degrees.

**Conclusions:**

Our findings highlight the need for greater policy attention to mental health inequalities between Internal migrants and those who never move away from their hometown in the United States. Our study provides a foundation for further research.

## Introduction

Depression and other mental health conditions are on the rise globally and are a public health concern; in particular, rates of depression and anxiety disorders are increasing [1]. Depression is a common mental disorder worldwide, and approximately 280 million people have depression [2]. Depression is a leading cause of disability worldwide and is a major contributor to the overall global burden of disease [3]. According to the Human Development Report [4], there were 740 million internal migrants worldwide in 2009. Studies have indicated that health affects a person’s migration propensity, although the signs and extent of this effect are not obvious [5]. A study also found that internal migration has a significant negative impact on mental health [6]. Internal migration is a complex process that requires maximizing opportunities, satisfying individual aspirations or weathering difficult times [7]. Despite well-documented declines in mobility, the United States has a high level of population mobility, with approximately 10 percent of its population moving every year and each individual experiencing an average of 11.7 moves during their lifetime [8]. Internal migrants may move due to financial pressures, and depression and anxiety are common mental illnesses among labour migrants [9]. Internal migrants can also disrupt familial norms of coresidence and geographical proximity, increasing the risk of loneliness [10]. Various internal migration factors (e.g., major life events, poverty, unsafe and stressful migration experiences, discrimination, neighbourhood factors, family reunification, linguistic isolation) and social support factors contribute to the onset of depressive symptoms [11]. However, few studies have explored the association of internal migration with depressive symptoms [11]. Public health researchers and mental health care providers need a more comprehensive understanding of the factors that contribute to poor mental health in internal migrant families.

The purpose of this study was to examine the association between residential migration experience and the presence of depressive symptoms. Studies have shown that those who moved within Mexico reported more anxiety, chronic fatigue, and pain [12, 13]. Disparities in the association between migration experiences and mental health among people of different ages and genders were also investigated. Given that females are more susceptible to mental health risk factors than males [14, 15], we hypothesized that the association of internal migration experience with depression is stronger in females. A Chinese study demonstrated that internal migration experience was associated with higher risks of depressive symptoms, especially among females and younger adults. Another study provided clear evidence of a strong relationship between internal address change in early years and poor mental health in adulthood [16, 17]. There is study show also an association between Different internal migration causes and the onset of depressive symptoms [18].

Accordingly, we proposed the following four hypotheses:

### Hypothesis 1 (H1)

*Internal migration experience is positively associated with depressive symptoms.*

### Hypothesis 2 (H2)

*The association between internal migration experience and depressive symptoms is more significant in females and young adults.*

### Hypothesis 3 (H3)

*The association between internal migration experience and depressive symptoms is more significant in participants who might move.*

### Hypothesis 4 (H4)

*Internal migration causes mediate the association of internal migration experience with depressive symptoms.*

## Methods

### Participants

This study analysed data from the Panel Study of Income Dynamics (PSID). The Panel Study of Income Dynamics (PSID) are the longest-running longitudinal household survey in the world. PSID data are panel data, including both cross-sectional (between individuals) and longitudinal data. The study began in 1968 with a nationally representative sample of over 18,000 individuals from 5,000 families in the United States [19]. Information on these individuals and their descendants has been collected continuously, including data on employment, income, wealth, expenditures, health, marriage, parity, child development, philanthropy, education, and numerous other topics [19]. It also collected detailed information about internal migration and the reasons for migration among people over 18 years of age; thus, these data are ideal for our study purposes [19]. To understand the recent status of depressive symptoms, we used the 2005 to 2019 waves of the PSID, where all heads/references were asked about their internal migration experience and depressive symptoms, including 69,218 family units (FUs) (or observations). First of all, we extracted only data from the first time the participants (n = 15,023) answered the questionnaire; recent home visitors or international migrants were not included in the study. For these participants, we performed exploratory analyses, t tests or chi-square tests univariate analysis, multivariate regression analysis. Second, we used a fixed effects model to analyze the PSID panel data from 2005 to 2019. The PSID followed respondents biennially from 2005 to 2019. According to our study purpose, “head/reference” was selected as the research object.

### Measures

#### Internal migration

Internal migration refers to long-distance moves, local moves or short-distance mobility within counties or among counties, cities, or states [8]. Since the United States is a country of immigrants, the PSID sample includes both indigenous people and immigrants; however, this study only included individuals who had settled in the United States for more than eight years. The inclusion criteria for internal immigrants in this study were as follows.

Internal migration experience was determined from the “Residence” section of the PSID. We used the related measures of internal migration: if the FU had ever left their place of residence, moving across state boundaries but within national boundaries, for at least 6 months, which is often considered the threshold of migration or changing of the usual residence [20], then the participant was considered to have internal migration experience, and the corresponding response was assigned to “Yes” (variable setting: Yes = 1). If the FU had never moved from their place of residence to a new location for 6 months or longer, the participant was considered to have no internal migration experience, and the corresponding response was assigned to “No” (variable setting: No = 0).

Aside from questions related to whether the FU moved to a different housing unit, information was also collected on migration experience during childhood, plans to move in future intervals and basic reasons for moving. Migration experience during childhood was measured by dichotomous variables (yes = 1, no = 0), and plans to move in the future were also measured by dichotomous variables (yes = 1, no = 0). Basic reasons for moving were categorized as follows: “has not moved”, “to take another job; transfer; stopped going to school”, “to get nearer to work”, “more space; more rent; better place”, “less space; less rent”, “get own home/place; got married; physical conditions of the previous housing unit”, “better neighborhood; go to school; to be closer to friends and/or relatives”, “housing unit (HU) coming down; being evicted; armed services, etc.; health reasons; divorce; retiring because of health”, “ambiguous, mixed, or other reasons, including reasons such as to save money, all my old neighbors moved away, retiring” and “homeless” (variable setting: 0 to 9).

#### Depressive symptoms

The information on depressive symptoms was collected in the health module of the PSID. Depressive symptoms were assessed using two components: [1] “What was the diagnosis? What is the emotional/psychiatric disorder?”, and he questions consists of three mentions [2]. The K-6 Non-Specific Psychological Distress Scale. And this measure has shown excellent specificity and sensitivity for depressive diagnoses [21–24]. There are also studies that have been shown the optimal cut-off value of K6 to detect any depressive/anxiety disorders is ≥ 12 (sensitivity 81.0%; specificity 76.6%) [23]. Therefore, we listed participants who had been diagnosed with depression in the past and K6 ≥ 12 as having depressive symptoms (variable setting: No = 0, Yes = 1).

#### Control variables

Previous studies have found that sex [1, 25, 26], age [25–27], marital status [26–28], developmental environment [29], and health-related quality of life [30–32], among others, are influencing factors of depressive symptoms. In the present study, the control variables in our analyses were most basic demographic characteristics, including individual, household, and personal life history characteristics. Individual characteristics included age (18 to 44 years old = 1, 45 to 59 years old = 2 and 60 years old and older = 3), sex (male = 1, female = 2) and marital status (married = 1, single = 2), residential location while growing up (farm/country = 1, small town/suburb = 2, large city = 3, other = 4) and religious (yes = 1). Family size (number of family members) and household income levels [33] (≤ 130% of the federal poverty level (FPL) = 1, > 130% to ≤ 350% of FPL = 2 and > 350% of FPL = 3) was the household characteristic, and difficulty in activities of daily living (ADLs) (yes = 1) was the personal life history characteristic. Difficulty in ADLs was assigned to “Yes” (variable setting: Yes = 1) if a participant reported difficulty in performing any of the following six tasks: eating, bathing, dressing, toileting, transferring (e.g., getting into or out of bed, lifting), and continence (control of urination and defecation) [34, 35]. These variables were controlled to exclude the influence of underlying factors or conditions in this study.

### Statistical analysis

To analyse participant characteristics, we first pooled the data from all years and conducted a descriptive analysis for all variables used in this study [36]. For each variable, exploratory analyses were performed to determine whether the data were normally distributed. T tests or chi-square tests were employed to compare data from participants with and without internal migration experience. We performed a univariate analysis of the control variables to identify variables that significantly differed according to migration status and then performed a multivariate regression analysis including only the significant variables from the univariate analysis.

We conducted multivariate regression analyses to determine the association between internal migration experiences and depressive symptoms (Model 1). To clearly compare models containing different individual characteristics or different early life conditions and to determine the relationship between depressive symptoms and migration, we analysed this relationship in subgroups stratified by sex, age group and so on. Specifically, the subgroups included males (Model 2), females (Model 3), young adults (18 to 44 years old; Model 4), middle-aged adults (45 to 59 years old; Model 5), older adults (60 years old and older; Model 6), participants who moved during childhood (Model 7), and participants who might move in the future (Model 8). The multiple logistic regression model was specified as follows:

$$\mathrm{ln}\left(\frac{{\pi }_{it}}{1-{\pi }_{it}}\right)={\beta }_{0}+{\beta }_{1}{Mig}_{it}+{\gamma }_{1}{X}_{1it}+\dots +{\gamma }_{k}{X}_{kit}$$

${\pi }_{it}$: the probability of having depressive symptoms in period *t* for the *i*th participant.

$1-{\pi }_{it}$: the odds of not having depressive symptoms in period *t* for the *i*th participant.

${Mig}_{it}$: a dummy variable that indicates whether a participant has internal migration experience (participants without any migration experience served as the reference group).

${X}_{1it}\dots {X}_{kit}$: a set of control variables, including individual, household, and personal life history characteristics.

We treated participants without any migration experience as the reference group. To better interpret the results, we present odds ratios (ORs) indicating how strongly the presence of depressive symptoms was associated with internal migration experience. In addition, we conducted a robustness check. To test the robustness of our main findings to the structure of the panel data, we extracted subgroups of participants interviewed and followed from 2005 to 2019 (panel data).

Panel data model selection: fixed effect with time. This study considered static panel analysis with White and Hausman tests to determine the final model. We used the White and Hausman tests to determine the model without control variables. Significant *P* < 0.05 indicated fixed effects.

The integration, processing and statistical analysis of the databases were performed using Stata version 17. The significance level for hypothesis testing was set to 0.05.

## Results

A total of 15,023 adults were included in the analyses, of whom 50.66% had internal migration experience 66.85% were aged 18 to 44 years, 21.03% were aged 45 to 59 years, 12.12% were aged 60 years or older, and 64.83% were men. In the full sample, approximately 48.03% were married. Other characteristics of the participants are shown in Table 1.

**Table 1:** Summary characteristics of the participants

Variable	Full sample (*n* = 15,023)	Internal migrants (*n* = 7,611)	Nonmigrants (*n* = 7,412)	*P* value (Chi-square test/t test)
**Depressive symptoms**				
No, n (%)	14,359 (95.58%)	7,240 (95.13%)	7,119 (96.05%)	0.006
Yes, n (%)	664 (4.42%)	371 (4.87%)	293 (3.95%)	
**Sex**				
Male, n (%)	9,740 (64.83%)	4,733 (62.19%)	5,007 (67.55%)	< 0.001
Female, n (%)	5,283 (35.17%)	2,878 (37.81%)	2,405 (32.45%)	
**Age (years)**				
18 to 44, n (%)	10,043 (66.85%)	6,557 (86.15%)	3,486 (47.97%)	< 0.001
45 to 59, n (%)	3,159 (21.03%)	785 (10.31%)	2,374 (31.33%)	
60 or older, n (%)	1,821 (12.12%)	269 (3.53%)	1,552 (20.70%)	
**Marital status**				
Married, n (%)	7,215 (48.03%)	3,167 (41.61%)	4,048 (54.61%)	< 0.001
Single, n (%)	7,808 (51.97%)	4,444 (58.39%)	3,364 (45.39%)	
**Residential location while growing up**				
Farm/country, n (%)	1,686 (11.52%)	521 (7.00%)	1,165 (16.21%)	< 0.001
Small town/suburb, n (%)	6,878 (47.01%)	3,630 (48.75%)	3,248 (45.20%)	
Large city/other, n (%)	6,068 (41.47%)	3,295 (44.25%)	2,773 (38.59%)	
**Moved during childhood**				
No, n (%)	6,082 (83.62%)	4,047 (81.96%)	2,035 (87.15%)	< 0.001
Yes, n (%)	1,191 (16.38%)	891 (18.04%)	300 (12.85%)	
**Might move**				
No, n (%)	7,633 (52.13%)	2,846 (38.38%)	4,787 (66.26%)	< 0.001
Yes, n (%)	7,008 (47.87%)	4,570 (61.62%)	2,438 (33.74%)	
**Having difficulty in ADLs**				
No, n (%)	13,548 (90.73%)	7,060 (93.35%)	6,488 (88.03%)	< 0.001
Yes, n (%)	1,385 (9.27%)	503 (6.65%)	882 (11.97%)	
**Family size (number of family members), mean (SD)**	2.75 (1.52)	2.64 (1.50)	2.86 (1.54)	< 0.001
**income levels**				
≦130% of FPL, n (%)	3,709 (24.69%)	2,222 (29.19%)	1,487 (20.06%)	< 0.001
> 130% to ≦350% of FPL, n (%)	6,026 (40.11%)	3,166 (41.60%)	2,860 (38.59%)	
> 350% of FPL, n (%)	5,288 (35.20%)	2,223 (29.21%)	3,065 (41.35%)	
**Religious**				
No, n (%)	2,531 (20.93%)	1,177 (20.07%)	1,354 (21.73%)	0.025
Yes, n (%)	9,564 (79.07%)	4,687 (79.93%)	4,877 (78.27%)	
**Year of survey**				
2005, n (%)	7,750 (51.59%)	2,673 (35.12%)	5,077 (68.50%)	< 0.001
2007, n (%)	1,363 (9.07%)	923 (12.13%)	440 (5.94%)	
2009, n (%)	1,110 (7.39%)	775 (10.18%)	335 (4.52%)	
2011, n (%)	1,094 (7.28%)	763 (10.02%)	331 (4.47%)	
2013, n (%)	1,033 (6.88%)	701 (9.21%)	332 (4.48%)	
2015, n (%)	999 (6.65%)	665 (8.74%)	334 (4.51%)	
2017, n (%)	856 (5.70%)	584 (7.67%)	272 (3.67%)	
2019, n (%)	818 (5.44%)	527 (6.92%)	291 (3.93%)	

Notes: *SD* Standard deviation, *ADL* Activities of daily living, *FPL* Federal Poverty Level

Table 1 presents the summary characteristics of the full sample and the two groups according to internal migration experience. In the full sample, the prevalence of depressive symptoms was 4.42%. Without considering the confounding effects of control variables, participants with internal migration experience (*n* = 7,611) were more likely to suffer from depressive symptoms (4.87% vs. 3.95%, *p* < 0.05) than those without internal migration experience (*n* = 7,412). In addition, participants with internal migration experiences were more likely to be female (37.81% vs. 32.45%, *p* < 0.001), be younger (86.15% vs. 47.97%, *p* < 0.001), be single (58.39% vs. 45.39%, *p* < 0.001), have grown up in a small city (48.75% vs. 45.20%, *p* < 0.001), have moved during childhood (18.04% vs. 12.85%, *p* < 0.001), have a possibility of moving again (61.62% vs. 33.74%, *p* < 0.001), have a smaller family (2.64 vs. 2.86, *p* < 0.001), income levels ≦130% of FPL (29.19% vs. 20.06%, *p* < 0.001), income levels > 130% to ≦350% of FPL (41.60% vs. 38.59%, *p* < 0.001) and be religious (79.93% vs. 78.27%, *p* < 0.05). In comparison with migrants, nonmigrants had higher levels of difficulty in ADLs (*p* < 0.001).

Table 2 shows the distribution of depression among participants with different migration causes. Migratory causes had a significant impact on depression (*χ*^2^ = 63.20, *p* < 0.001). More specifically, there was a statistically significant difference in the association between some migration causes (“Less space; less rent”; “Better neighborhood; go to school; to be closer to friends and/or relatives”; “HU coming down; being evicted; armed services, etc.; health reasons; divorce; retiring because of health”; and “Ambiguous, mixed, or other reasons, including reasons such as to save money, all my old neighbors moved away, retiring”) and depression symptoms (*p* < 0.05).

**Table 2:** The relationship between migration causes and depression

Migration cause	n	%	*χ*^2^	*P*
**No migration experience**				
	265	3.65%	63.20^a^	< 0.001^a^
**To take another job; transfer; stopped going to school**				
	25	5.11%	2.81	0.093
**To get nearer to work**				
	9	2.59%	0.98	0.323
**More space; more rent; better place**				
	46	3.70%	0.07	0.796
**Less space; less rent**				
	36	8.55%	23.34	< 0.001
**Get own home/place; got married; physical conditions of the previous housing unit**				
	102	4.20%	1.17	0.280
**Better neighborhood; go to school; to be closer to friends and/or relatives**				
	36	5.70%	6.01	0.014
**HU coming down; being evicted; armed services, etc.; health reasons; divorce; retiring because of health**				
	67	8.25%	37.99	< 0.001
**Ambiguous, mixed, or other reasons, including reasons such as to save money, all my old neighbors moved away, retiring**				
	67	5.01%	5.14	0.023
**Homeless**				
	3	2.11%	1.02	0.313

Notes: *HU* Housing unit

^a^ Results of a chi-square test for all migration causes

Table 3 shows the association between internal migration experience and depressive symptoms in terms of multivariate logistic regression analysis using the PSID data. The results showed that internal migration experience was associated with depressive symptoms, and the risk of depression in internal migrants was 1.259 times (OR = 1.259, 95% CI = (1.025–1.547, *p* < 0.01) (Model 1) that in nonmigrants after adjusting for sex, age, marital status, religious belief, family size, household income levels, possibility of moving, difficulty in ADLs and so on. These results support Hypothesis 1. In terms of sex differences, internal migration experience was significantly positively associated with female depressive episodes (OR = 1.312, 95% CI = 1.010–1.704, *p* < 0.05) (Model 3) but not with male depressive episodes (Model 2). In terms of age differences, internal migration experience significantly increased the risk of being depressed the in young individuals (OR = 1.304, 95% CI = 1.010–1.684, *p* < 0.05), which supports Hypothesis 2. In terms of the possibility of moving, compared to participants unlikely to move, respondents likely to move had significantly higher risks of developing depressive symptoms (OR = 1.459, 95% CI = 1.094–1.947, *p* < 0.01) (Model 8), which supports Hypothesis 3.

**Table 3:** Overall and stratified association between migration experience and depressive symptoms

	Model 1	Model 2	Model 3	Model 4	Model 5	Model 6	Model 7	Model 8
	Overall	Men	Women	Age: 18 ~ 44	Age: 45 ~ 60	Age ≥ 60	Moved during childhood	Might move
	OR (95% CI)	OR (95% CI)	OR (95% CI)	OR (95% CI)	OR (95% CI)	OR (95% CI)	OR (95% CI)	OR (95% CI)
**Internal migration experience**								
No	Ref	Ref	Ref	Ref	Ref	Ref	Ref	Ref
Yes	1.259 (1.025–1.547) **	0.930 (0.657–1.317)	1.312 (1.010–1.704) *	1.304 (1.010–1.684) *	1.389 (0.938–2.056)	0.963 (0.458- 2.023)	1.285 (0.990–1.669)	1.459 (1.094–1.947) **
**Control variables**	Yes	Yes	Yes	Yes	Yes	Yes	Yes	Yes
**R-squared** **/Pseudo-R-** **squared**	0.046	0.039	0.047	0.044	0.055	0.050	0.046	0.042

Notes: *OR* Odds ratio, *CI* Confidence interval, *Ref*. Reference group. The Wald test (Z statistic) was performed to check statistical significance; * *p* < 0.05, ** *p* < 0.01

Table 4 shows the association of depressive symptoms with internal migration causes (Model 9). The results demonstrated that “Less space; less rent” (OR = 2.441, 95% CI = 1.281–4.653, *p* < 0.01) and “HU coming down; being evicted; armed services, etc.; health reasons; divorce; retiring because of health” (OR = 2.024, 95% CI = 1.125–3.641, *p* < 0.05) were associated with a significantly higher risk of having depressive symptoms among internal migrants, while other migration causes did not differ in the risk of depression, which supports Hypothesis 4.

**Table 4:** Association between internal migration experience and depressive symptoms by internal migration causes

	Model 9
	OR (95% CI)
**Causes of internal migration experience**	
No migration experience	Ref
To take another job; transfer; stopped going to school	1.774 (0.883–3.564)
To get nearer to work	0.784 (0.299–2.051)
More space; more rent; better place	1.445 (0.788–2.651)
Less space; less rent	2.441 (1.281–4.653) **
Get own home/place; got married; physical conditions of the previous housing unit	1.374 (0.778–2.425)
Better neighborhood; go to school; to be closer to friends and/or relatives	1.588 (0.830–3.041)
HU coming down; being evicted; armed services, etc.; health reasons; divorce; retiring because of health	2.024 (1.125–3.641) *
Ambiguous, mixed, or other reasons, including reasons such as to save money, all my old neighbors moved away, retiring	1.438 (0.808–2.560)
Homeless	0.951 (0.268–3.368)
**Control variables**	Yes
**R-squared/Pseudo-R-squared**	0.096

Notes: *OR* Odds ratio, *CI* Confidence interval, *Ref*. Reference group, *HU* Housing unit. The Wald test (Z statistic) was performed to check statistical significance; * p < 0.05, ** p < 0.01

Table 5 presents robustness checks of the association between migration experience and depressive symptoms. We extracted panel data from 2005 to 2019 and re-estimated the overall sample. Internal migration experience in Model 10 was significantly (*p* < 0.05) and positively (β = 0.006) associated with depressive symptoms. Internal migration experience in Model 11 was also significantly (*p* < 0.05) and positively (β = 0.007) associated with depressive symptoms. In summary, the results suggest that our main findings are robust to the structure of the panel data.

**Table 5:** Results of fixed effects with time

	Model 10	Model 11
	Depressive symptoms (no control variables)	Depressive symptoms (within control variables)
	Coefficient (Std. err.)	Coefficient (Std. err.)
**Internal migration experience**	0.006 (0.003) *	0.007 (0.003) *
**R-squared/Pseudo-R-squared**	0.0019	0.0123
**Number of observations**	23,360	18,872
**F**	5.28	8.33
**Prob(F-statistic)**	0.0003	0.0000

Notes: * *p* < 0.05

## Discussion

In large population-based cohorts from the PSID, we found evidence that internal migration experience was associated with depressive symptoms. And internal migration experience is positively associated with depressive symptoms, which is consistent with the research by *Familiar I *[13] and *Zheng X *[37]. This finding is also consistent with findings in countries such as Mexico [12], Indonesia [38], England [39] and Peru [18, 40], showed internal migration experience to be a risk factor against depressive symptoms.

There were sex differences in the association between migration and depressive symptoms. Internal migration experience was significantly positively associated with female depressive episodes but not with male depressive episodes, which is consistent with the research by *Donato KM *[12]. Some studies also suggest that migration is associated with positive labour market outcomes for married men but negative labour market outcomes for women [41, 42]. Our findings underscore the necessity of improving psychosocial counselling and providing the necessary social and emotional support for internal migrants, especially for women. Compared with adults of other ages, young people (aged 18 to 44) with internal migration experience are more likely to have depressive symptoms. This may be related to changes in the social status and role of young people after employment. The activities of people in this age group are mainly colleague-based, which is accompanied by enlarging of the field of life, the scope of activities and interpersonal socialization, which can easily lead to poor mental health. A feasible explanation is that, young migrants young (aged 18 to 44) need to take on more family financial responsibilities when they first migrate to cities, such as children’s educational expenditure and economic support for elderly parents [43].

The results of our study are consistent with those of analysis by Tunstall H and Green MA, which showed evidence of a positive association between poor health and internal mobility as well as internal migration. Our research shows that internal migration experience was significantly and positively linked to depressive symptoms among participants who might move. We found that specific migration causes (“Less space; less rent” and “HU coming down; being evicted; armed services, etc.; health reasons; divorce; retiring because of health”) significantly increased the risk of depressive symptoms. At the same time, the migration causes should be explored by local authorities, and some social and economic assistance should be provided, if necessary, to reduce internal migration. It is equally important to develop interventions that are evidence based, such as reducing employment discrimination and racial discrimination and equal distribution of health resources. It is important to improve psychological health among internal migrants.

Internal migration is a source of potential specific stressors that could threaten migrants' mental health [44], but the number of migrants has been steadily increasing in recent years [45]. Notably, this relationship with depression (a common mental illness worldwide) has been confirmed by many researchers. Our study also provides evidence for further research, such as exploring the interaction of certain factors that may mediate the association between internal migration experience and depressive symptoms.

Our analyses have several limitations. First, although we used participant self-report and K6 scale, which is highly reliable in measuring depression, measurements to diagnose depression, it does not provide a clinical diagnosis of depression. Second, due to the data constraints, Second, due to the data constraints, we first extracted data from participants who responded to the questionnaire for the first time, so the analysed data were cross-sectional studies. Therefore, the analysis of this study should be interpreted as association rather than causal inference. Third, for fixed-effect analyses, we extracted only panel data from 2009 to 2017 for analysis in order to balance the data as they were unbalanced panels. Finally, our sample included only adults aged 18 years and older, who do not represent underage internal migrants, suggesting that our findings should be interpreted and generalized with caution.

## Conclusions

Overall, we found that depressive symptoms in adults were associated with the experience of internal migration. Our findings highlight the need for greater policy attention to mental health inequalities between Internal migrants and those who never move away from their hometown in the United States. To address the mental health issues faced by migrants from the mainland, policies must address broader social determinants, such as income and employment status.

## Data Availability

The data underlying the results presented in the study are available from the Panel Study of Income Dynamics (PSID Home (umich.edu)), public use dataset.

## Abbreviations

PSID: The Panel Study of Income Dynamics
FU: Family unit
OR: Odds ratio
PHQ-2: 2-Item Patient Health Questionnaire
HU: Housing unit
FPL: Federal poverty level;
ADLs: Activities of daily living
SD: Standard deviation
CI: Confidence interval
Ref.: Reference group
